# Supplementary material for: Critical couplings in topological-insulator waveguide-resonator systems observed in elastic waves
Source: Natl Sci Rev. 2020 Oct 23;8(2):nwaa262. doi: 10.1093/nsr/nwaa262 (PMC8288343; doi:10.1093/nsr/nwaa262)
Supplement: nwaa262_Supplement_File [file nwaa262_supplement_file.pdf]

Supplementary Information

for

Critical couplings in topological insulator waveguide-resonator systems observed in elastic waves

Si-Yuan Yu[†1,2,3*], Cheng He[†1,2,3], Xiao-Chen Sun[†1], Ji-Qian Wang[1], Zi-Dong Zhang[1], Bi-Ye Xie[1], Hong-Fei Wang[1], Yuan Tian[1], Ming-Hui Lu[1,2,3*], and Yan-Feng Chen[1,2,3*]

[1]*National Laboratory of Solid State Microstructures & Department of Materials Science and Engineering, Nanjing University, Nanjing, Jiangsu 210093, China.*

[2]*Collaborative Innovation Center of Advanced Microstructures, Nanjing University, Nanjing, Jiangsu 210093, China.*

[3]*Jiangsu Key Laboratory of Artificial Functional Materials, Nanjing University, Nanjing 210093, China*

[†]*These authors contributed equally to this work.*

**e-mail: yusiyuan@nju.edu.cn; luminghui@nju.edu.cn; yfchen@nju.edu.cn.*

This Supplementary Information includes:

➢ **Supplementary Note I: Microscopic Theory for the TI Cavity**

——Part I: Paired Degenerate Eigenstates

（Supplementary Figs. 1 and 2）

——Part II: Paired Eigenstates with Broken Degeneracy

（Supplementary Fig. 3 & Tables S1, S2, and S3）

➢ **Supplementary Note II: Other Supplements for the Experiments**

——Supplementary Fig. 4 | Structures of elastic OI and TI used in this research

——Supplementary Fig. 5 | TI cavities of different sizes and their eigenmodes

——Supplementary Fig. 6 | Paired cavity modes shown in Fig. 1 of the main text

——Supplementary Fig. 7 | Mini-gap of the two elastic helical edge states at $k_l = 0$

——Supplementary Fig. 8 | Photograph of the front of one of our experimental samples

——Supplementary Fig. 9 | Cavity mode excitations of two different categories

——Supplementary Fig. 10 | TI waveguide-resonator distance for critical couplings

——Supplementary Fig. 11 | Experiment vs. simulation results for TI waveguide-resonator critical couplings

Supplementary Note I: Microscopic Theory for the TI Cavity

——Part I: Paired Degenerate Eigenstates

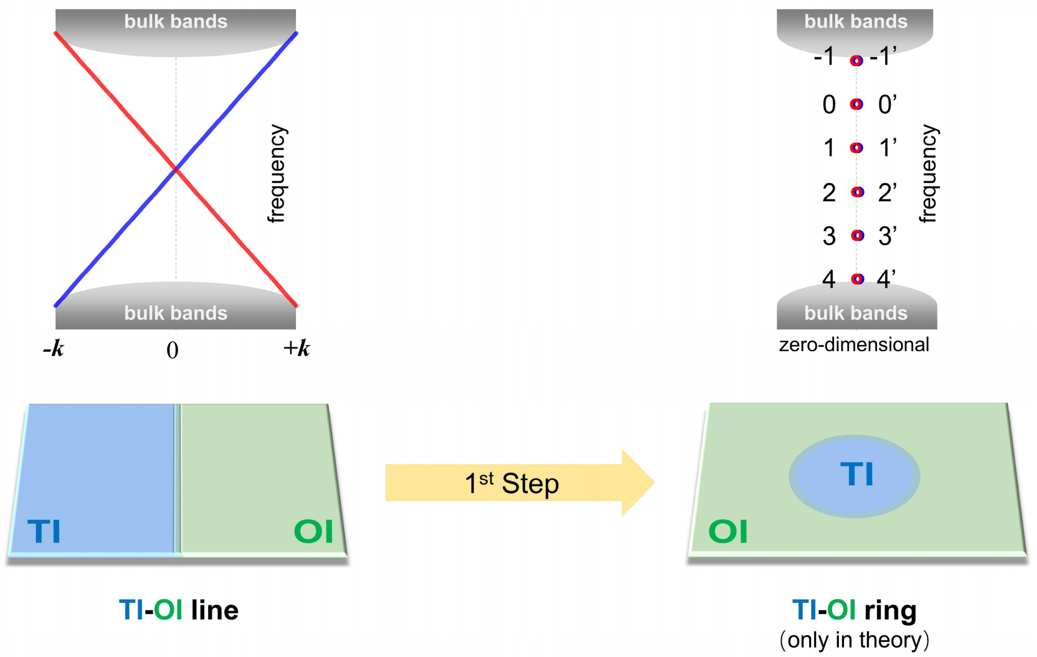

Supplementary Fig. 1 | From 2D helical edge states to paired degenerate eigenstates

In our implementation, if we choose the basis (p_+, d_+, p_-, d_-), then the effective $k{\cdot}p$ Hamiltonian is expressed as

$$H = \begin{bmatrix} M+Bk^2 & Ak_+ & & \\ A^*k_- & -M-Bk^2 & & \\ & & M+Bk^2 & Ak_- \\ & & A^*k_+ & -M-Bk^2 \end{bmatrix}, \tag{S1}$$

where $k_\pm = k_x \pm ik_y$ and $k^2 = k_x{}^2 + k_y{}^2$. $A = -i|A|$ is a purely imaginary number. B and M are real numbers. We next consider a finite-sized sample with circular geometry. Under polar coordinates, we have

$$\begin{aligned} k_x &= \cos\phi k_r - \sin\phi k_\phi \\ k_y &= \sin\phi k_r + \cos\phi k_\phi \end{aligned}, \tag{S2}$$

in which

$$k_x = -i\partial_x, k_y = -i\partial_y, k_r = -i\partial_r, k_\phi = (1/r)(-i\partial_\phi), \tag{S3}$$

such that

$$k^2 = k_x^2 + k_y^2 = -(\partial_x^2 + \partial_y^2) = -\left(\partial_r^2 + \frac{1}{r^2}\partial_\phi^2 + \frac{1}{r}\partial_r\right), \tag{S4}$$

$$k_{\pm} = k_x \pm ik_y = \left(\cos\phi k_r - \sin\phi k_\phi\right) \pm i\left(\sin\phi k_r + \cos\phi k_\phi\right) = e^{\pm i\phi} k_r \pm ie^{\pm i\phi} k_\phi . \quad \textbf{(S5)}$$

Our discussion focuses on the interface, leading to $r \to R$, where R is the position of the interface, as shown in **Supplementary Fig. 2**. Noting that the wave decays from the interface, we assume that $k_r = -i\partial_r \to -i\kappa, \kappa \in \mathbb{R}$ and $\kappa \gg 1/R$, where κ is the decay rate of the field.

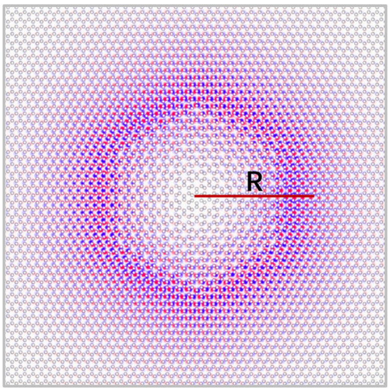

Supplementary Fig. 2 | Field distribution of a representative topological cavity eigenstate.

We therefore ignore the term $\frac{1}{R^2}$ and assume

$$k^2 = -\left(\partial_r^2 + \frac{1}{r^2}\partial_\phi^2 + \frac{1}{r}\partial_r\right) \to -\left(\kappa^2 + \frac{1}{R}\kappa + \frac{1}{R^2}\partial_\phi^2\right) \approx -\left(\kappa^2 + \frac{1}{R}\kappa\right) \to -\left(\partial_r^2 + \frac{1}{R}\partial_r\right). \quad \textbf{(S6)}$$

Substituting Eqs. S2, S4, and S5 into Eq. S1 and separating the radial component of k from the transverse component, we have

$$H = \begin{bmatrix} M & Ae^{i\phi}k_r & & \\ A^* e^{-i\phi}k_r & -M & & \\ & & M & Ae^{-i\phi}k_r \\ & & A^* e^{i\phi}k_r & -M \end{bmatrix} + \begin{bmatrix} -B\left(\partial_r^2 + \frac{1}{R}\partial_r\right) & iAe^{i\phi}k_\phi & & \\ -iA^* e^{-i\phi}k_\phi & B\left(\partial_r^2 + \frac{1}{R}\partial_r\right) & & \\ & & -B\left(\partial_r^2 + \frac{1}{R}\partial_r\right) & -iAe^{-i\phi}k_\phi \\ & & iA^* e^{i\phi}k_\phi & B\left(\partial_r^2 + \frac{1}{R}\partial_r\right) \end{bmatrix}$$

. **(S7)**

The second part of Eq. S5 can be regarded as perturbations, and we neglect the small second-order amount. We have

$$H_0=\begin{bmatrix} M & Ae^{i\phi}k_r & & \\ A^*e^{-i\phi}k_r & -M & & \\ & & M & Ae^{-i\phi}k_r \\ & & A^*e^{i\phi}k_r & -M \end{bmatrix}, \tag{S8}$$

$$\Delta H=\begin{bmatrix} -B\left(\partial_r^2+\frac{1}{R}\partial_r\right) & iAe^{i\phi}k_\phi & & \\ -iA^*e^{-i\phi}k_\phi & B\left(\partial_r^2+\frac{1}{R}\partial_r\right) & & \\ & & -B\left(\partial_r^2+\frac{1}{R}\partial_r\right) & -iAe^{-i\phi}k_\phi \\ & & iA^*e^{i\phi}k_\phi & B\left(\partial_r^2+\frac{1}{R}\partial_r\right) \end{bmatrix}. \tag{S9}$$

With the basis (p_+,d_+,p_-,d_-) transformed to $(e^{i\phi}p_+,d_+,e^{-i\phi}p_-,d_-)$, the unperturbed Hamiltonian is

$$H_0=\begin{bmatrix} M & Ak_r & & \\ A^*k_r & -M & & \\ & & M & Ak_r \\ & & A^*k_r & -M \end{bmatrix}. \tag{S10}$$

In fact, because of the different topologies, the mass terms are different inside $\left(M(r<R)=+M_1>0\right)$ and outside $\left(M(r>R)=-M_2<0\right)$ the interface. Using the conditions $\psi(r\to R^-)=\psi(r\to R^+)$ and $\psi(r\to 0)=\psi(r\to\infty)=0$, we obtain the eigenvalue and eigenwave functions:

$$\omega_0=0 \tag{S11}$$

$$|1\rangle=\left(e^{i\phi}p_+ \quad d_+ \quad e^{-i\phi}p_- \quad d_-\right)\begin{pmatrix}1\\1\\0\\0\end{pmatrix}F(r)=\left(p_+ \quad d_+ \quad p_- \quad d_-\right)\begin{pmatrix}e^{i\phi}\\1\\0\\0\end{pmatrix}F(r), \tag{S12}$$

$$|2\rangle=\left(e^{i\phi}p_+ \quad d_+ \quad e^{-i\phi}p_- \quad d_-\right)\begin{pmatrix}0\\0\\1\\1\end{pmatrix}F(r)=\left(p_+ \quad d_+ \quad p_- \quad d_-\right)\begin{pmatrix}0\\0\\e^{-i\phi}\\1\end{pmatrix}F(r), \tag{S13}$$

with

$$F(r)=\sqrt{\frac{M_1M_2}{|A|(M_1+M_2)}}\begin{cases} e^{\frac{M_1}{|A|}(r-R)}, r<R \\ e^{-\frac{M_2}{|A|}(r-R)}, r>R \end{cases}, \tag{S14}$$

$$\int_0^\infty F(r)^2 \, dr = 1/2 \,. \tag{S15}$$

Considering the perturbations,

$$H = \begin{pmatrix} \langle 1|\Delta H|1\rangle & \langle 1|\Delta H|2\rangle \\ \langle 2|\Delta H|1\rangle & \langle 2|\Delta H|2\rangle \end{pmatrix} = \begin{pmatrix} |A|k_\phi + \frac{|A|}{2R} & 0 \\ 0 & |A|k_\phi - \frac{|A|}{2R} \end{pmatrix} \to \begin{pmatrix} |A|\frac{m}{R} + \frac{|A|}{2R} & 0 \\ 0 & -|A|\frac{m}{R} + \frac{|A|}{2R} \end{pmatrix}. \tag{S16}$$

We here quantize the wavevector k_ϕ :

$$k_\phi = \frac{-i\partial_\phi}{R} \to \frac{m}{R} \tag{S17}$$

and

$$\langle 1|\Delta H|1\rangle = \int_0^\infty dr \begin{pmatrix} e^{-i\phi} & 1 & 0 & 0 \end{pmatrix} F(r) \begin{bmatrix} B\left(\partial_r^2 + \frac{1}{R}\partial_r\right) & iAe^{i\phi}k_\phi & & \\ -iA^* e^{-i\phi}k_\phi & -B\left(\partial_r^2 + \frac{1}{R}\partial_r\right) & & \\ & & B\left(\partial_r^2 + \frac{1}{R}\partial_r\right) & -iAe^{-i\phi}k_\phi \\ & & iA^* e^{i\phi}k_\phi & -B\left(\partial_r^2 + \frac{1}{R}\partial_r\right) \end{bmatrix} \begin{pmatrix} e^{i\phi} \\ 1 \\ 0 \\ 0 \end{pmatrix} F(r)$$

$$= \int_0^\infty dr \left(F(r) B\left(\partial_r^2 + \frac{1}{R}\partial_r\right) F(r) - F(r) B\left(\partial_r^2 + \frac{1}{R}\partial_r\right) F(r) \right) + \left(\int_0^\infty F(r)^2 \, dr \right) \left(e^{-i\phi}\left(iAe^{i\phi}k_\phi\right) + \left(-iA^* e^{-i\phi}k_\phi\right)\left(e^{i\phi}\right) \right)$$

$$= \frac{1}{2}\left(iAk_\phi - iA^* k_\phi - i\frac{A^*}{R} \right) = |A|k_\phi + \frac{|A|}{2R}$$

$$. \tag{S18}$$

We finally obtain the eigenvalues

$$\boxed{\omega = \omega_0 + \frac{|A|}{R}\left(\frac{1}{2} + m\right) = \omega_0 + \frac{|A|}{R}\left(-\frac{3}{2} + n\right)}, \tag{S19}$$

with degenerate eigenstates

$$\begin{aligned} |m,+\rangle &= e^{im\phi}\left(e^{i\phi}p_+ + d_+\right)F(r) = e^{i(m+2)\phi}\left(e^{-i\phi}p_+ + e^{-i2\phi}d_+\right)F(r) = e^{in\phi}\left(e^{-i\phi}p_+ + e^{-i2\phi}d_+\right)F(r) = |n,+\rangle \\ |m,-\rangle &= e^{-im\phi}\left(e^{-i\phi}p_- + d_-\right)F(r) = e^{-i(m+2)\phi}\left(e^{i\phi}p_- + e^{i2\phi}d_-\right)F(r) = e^{-in\phi}\left(e^{i\phi}p_- + e^{i2\phi}d_-\right)F(r) = |n,-\rangle \end{aligned}. \tag{S20}$$

Passing along the interface, the state p_+ increases by 2π while the term $e^{-i\phi}$ increases by -2π ; *i.e.*, there is a balance in the term $e^{-i\phi}p_+$. The term $e^{-i\phi}p_+$ can be taken as not contributing any global angular momentum to the system, which is the same case as for the terms $e^{-i\phi}p_+$, $e^{-i2\phi}d_+$, $e^{i\phi}p_-$, and $e^{i2\phi}d_-$. Thus, the phase term outside the brackets in Eq. S19, $n = m + 2$, represents the total global angular momentum. In the case of standing waves, it is best to describe the parameter n as the number of nodes. The COMSOL

simulation results match the theoretical analysis, as shown in **Supplementary Fig. 5**.

——**Part II: Paired Eigenstates with Broken Degeneracy**

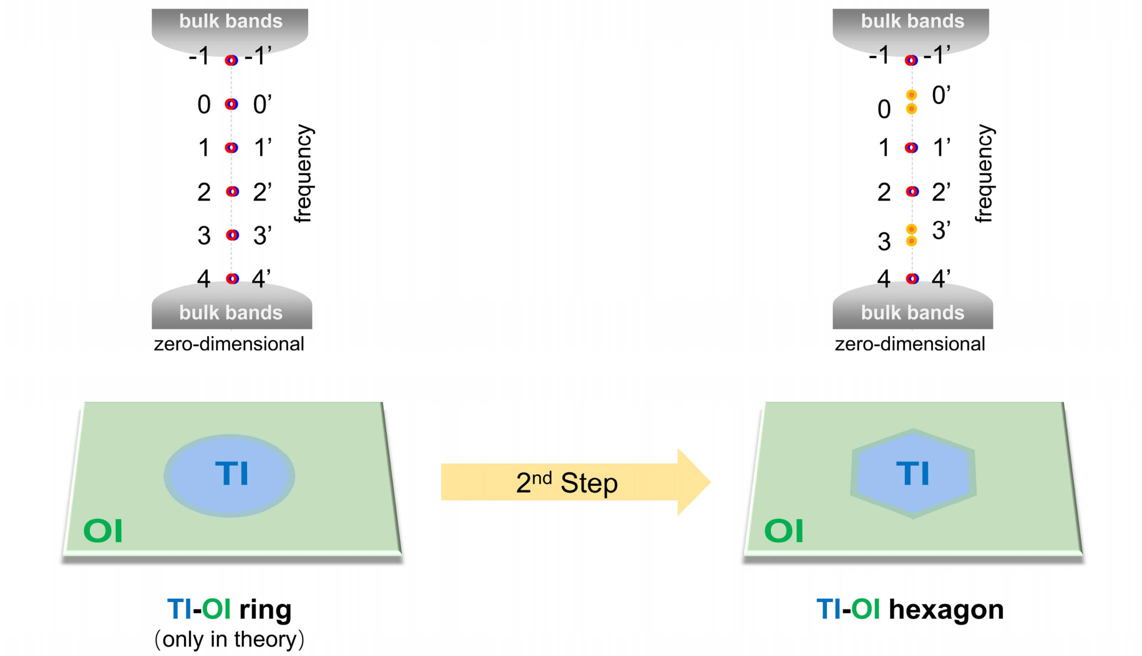

Supplementary Fig. 3 | Paired eigenstates with broken degeneracy

We previously considered a circular geometry of the finite lattice. We now consider a hexagonal geometry of the lattice that has C_{6v} symmetry. This rotational symmetry has a non-trivial effect on the eigenfunctions, as shown in Tables S1 and S2.

symmetry operators	$\begin{pmatrix} p_+ \\ p_- \end{pmatrix}$	$\begin{pmatrix} d_+ \\ d_- \end{pmatrix}$	$e^{i\phi}$
C_6	$\begin{pmatrix} e^{\frac{1}{3}\pi i} & 0 \\ 0 & e^{-\frac{1}{3}\pi i} \end{pmatrix}$	$\begin{pmatrix} e^{\frac{2}{3}\pi i} & 0 \\ 0 & e^{-\frac{2}{3}\pi i} \end{pmatrix}$	$\phi \to \phi + \frac{1}{3}\pi$
C_3	$\begin{pmatrix} e^{\frac{2}{3}\pi i} & 0 \\ 0 & e^{-\frac{2}{3}\pi i} \end{pmatrix}$	$\begin{pmatrix} e^{\frac{4}{3}\pi i} & 0 \\ 0 & e^{-\frac{4}{3}\pi i} \end{pmatrix}$	$\phi \to \phi + \frac{2}{3}\pi$
σ_y	$\begin{pmatrix} 0 & 1 \\ 1 & 0 \end{pmatrix}$	$\begin{pmatrix} 0 & 1 \\ 1 & 0 \end{pmatrix}$	$\phi \to -\phi$
σ_x	$\begin{pmatrix} 0 & -1 \\ -1 & 0 \end{pmatrix}$	$\begin{pmatrix} 0 & 1 \\ 1 & 0 \end{pmatrix}$	$\phi \to \pi - \phi$

Table S1 | Basis under C_{6v} symmetry operators.

symmetry operators	$\|n,+\rangle$ $= e^{in\phi}\left(e^{-i\phi}p_+ + e^{-i2\phi}d_+\right)$	$\|n,-\rangle$ $= e^{-in\phi}\left(e^{i\phi}p_- + e^{i2\phi}d_-\right)$	$\langle n,+\|$ $= e^{-in\phi}\left(e^{i\phi}p_- + e^{i2\phi}d_-\right)$	$\langle n,-\|$ $= e^{in\phi}\left(e^{-i\phi}p_+ + e^{-i2\phi}d_+\right)$
C_6	$e^{in\frac{\pi}{3}}\|n,+\rangle$	$e^{-in\frac{\pi}{3}}\|n,-\rangle$	$e^{-in\frac{\pi}{3}}\langle n,+\|$	$e^{in\frac{\pi}{3}}\langle n,-\|$
C_3	$e^{in\frac{2\pi}{3}}\|n,+\rangle$	$e^{-in\frac{2\pi}{3}}\|n,-\rangle$	$e^{-in\frac{2\pi}{3}}\langle n,+\|$	$e^{in\frac{2\pi}{3}}\langle n,-\|$
σ_y	$\|n,-\rangle$	$\|n,+\rangle$	$\langle n,-\|$	$\langle n,+\|$
σ_x	$e^{in\pi}\|n,-\rangle$	$e^{-in\pi}\|n,+\rangle$	$e^{-in\pi}\langle n,-\|$	$e^{in\pi}\langle n,+\|$

Table S2 | Eigenfunctions under C_{6v} symmetry operators.

We now apply perturbation theory to study the perturbed Hamiltonian arising from the coupling of up and down (pseudo)spins, which we denote $|n,+\rangle = |+\rangle$ and $|n,-\rangle = |-\rangle$. The perturbation Hamiltonian is $H = \begin{pmatrix} \langle +|H_s|+\rangle & \langle +|H_s|-\rangle \\ \langle -|H_s|+\rangle & \langle -|H_s|-\rangle \end{pmatrix} = \begin{pmatrix} H_{++} & H_{+-} \\ H_{-+} & H_{--} \end{pmatrix}$. The matrix elements of H are affected as shown in Table S3.

symmetry operators	H_{++}	H_{--}	H_{+-}	H_{-+}
C_6	H_{++}	H_{--}	$e^{-\frac{2}{3}n\pi i}H_{+-}$	$e^{\frac{2}{3}n\pi i}H_{-+}$
C_3	H_{++}	H_{--}	$e^{-\frac{4}{3}n\pi i}H_{+-}$	$e^{\frac{4}{3}n\pi i}H_{-+}$
σ_y	H_{--}	H_{++}	H_{-+}	H_{+-}
σ_x	H_{--}	H_{++}	H_{-+}	H_{+-}

Table S3 | Matrix elements of H under C_{6v} symmetry operators.

The Hamiltonian H should be unchanged under symmetry operations such that

$$\boxed{\begin{aligned} &H_{++} = H_{--} \\ &H_{+-} = H_{-+} \\ &H_{+-} = e^{-\frac{2}{3}n\pi i}H_{+-} = e^{-\frac{4}{3}n\pi i}H_{+-} \end{aligned}} \quad . \tag{S21}$$

We first see that the diagonal and off-diagonal terms of H are the same. The off-diagonal terms vanish for the

states with $n = 3k \pm 1$ (*e.g.*, $n = 1, 2, 4, 5, \ldots$), which means that the perturbations from the six corners balance each other and these states maintain degeneracy. However, for the states with $n = 3k$ (*e.g.*, $n = 0, 3, 6, \ldots$), the perturbations from the shape changes at the six corners do not balance, and the degeneracy is broken for these states.

For the states with $n = 3k$, we directly obtain the perturbed eigenfunctions:

$$\begin{aligned} \psi_1 &= \frac{1}{\sqrt{2}}\begin{pmatrix} 1 & 1 \end{pmatrix}\begin{pmatrix} |+\rangle \\ |-\rangle \end{pmatrix} \\ \psi_2 &= \frac{1}{\sqrt{2}}\begin{pmatrix} 1 & -1 \end{pmatrix}\begin{pmatrix} |+\rangle \\ |-\rangle \end{pmatrix} \end{aligned}. \tag{S22}$$

As an example, when $n = 0$, we have

$$\begin{aligned} \psi_1 &= p_x \cos\phi + p_y \sin\phi + d_{x^2-y^2} \cos 2\phi + d_{2xy} \sin 2\phi \\ \psi_2 &= p_y \cos\phi - p_x \sin\phi + d_{2xy} \cos 2\phi - d_{x^2-y^2} \sin 2\phi \end{aligned}, \tag{S23}$$

and when $n = 3$, we have

$$\begin{aligned} \psi_1 &= p_x \cos 2\phi - p_y \sin 2\phi + d_{x^2-y^2} \cos\phi - d_{2xy} \sin\phi \\ \psi_2 &= p_y \cos 2\phi + p_x \sin 2\phi + d_{2xy} \cos\phi + d_{x^2-y^2} \sin\phi \end{aligned}. \tag{S24}$$

All these eigenfunctions are real functions, which means that the waves are standing waves.

For other states with $n = 3k \pm 1$, the degeneracies are maintained, and the eigenfunctions have both real parts and imaginary parts, representing propagating waves.

Supplementary Note II: Other Supplements for the Experiments

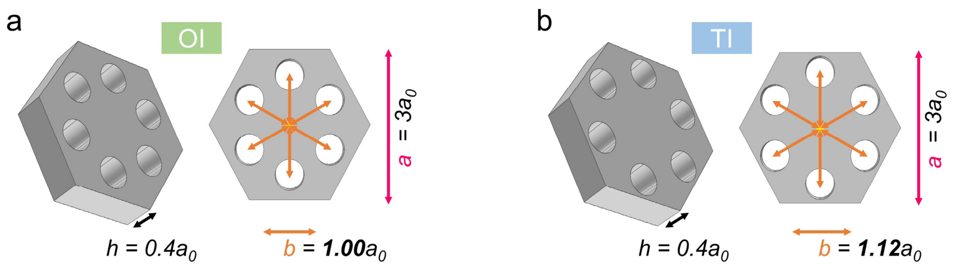

Supplementary Fig. 4 | Structures of elastic OI and TI used in this research.

a: Unit cell of an elastic OI and **b**: unit cell of a TI. The TI and OI have the same lattice constant (a=3a_0) in the hexagonal lattice, and each unit cell contains six of the same perforated holes in a plain plate with the same thickness. The only difference is the hole-centre distance, b, which equals a_0 (OI) and 1.12 a_0 (TI).

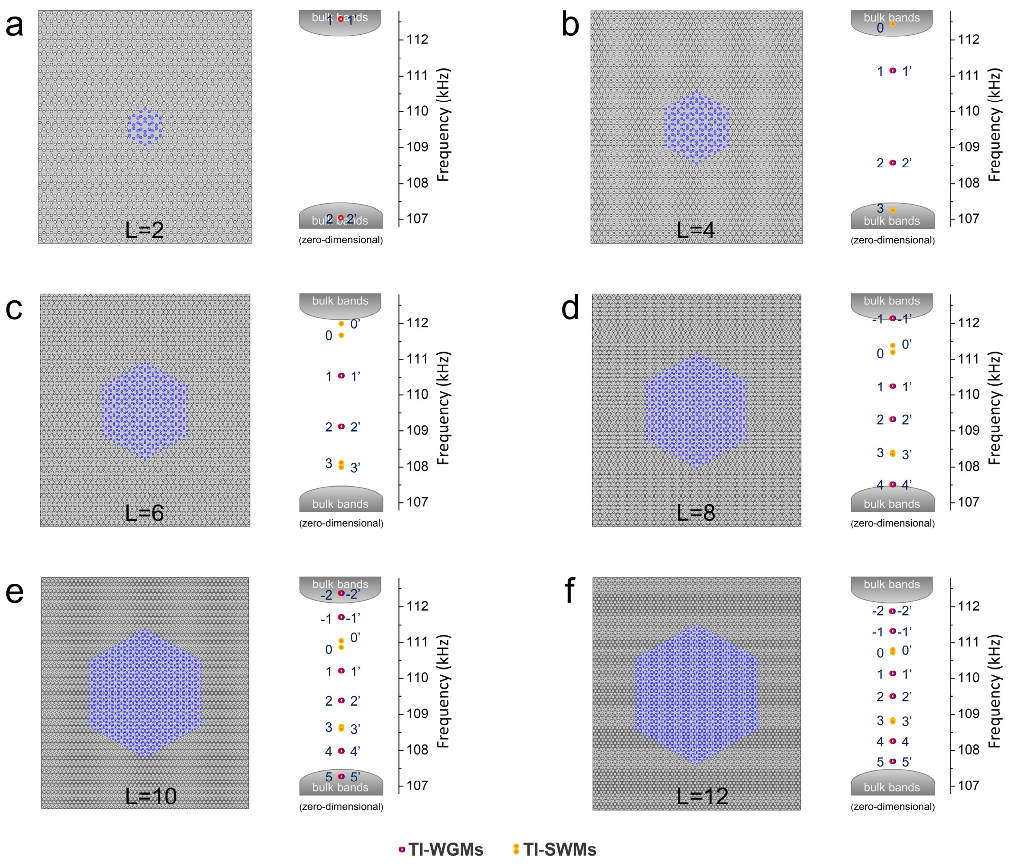

Supplementary Fig. 5 | TI cavities of different sizes and their eigenmodes.

For convenience of research, we set the shape of the TI particles to a regular hexagon, the size of which depends only on a single parameter, *i.e.*, the side length. When the side length is from two lattice constants to twelve lattice constants (L from 2 to 12), the cavity modes appearing in the bulk band gap of the TI/OI are as shown in **a** to **h**, respectively. No cavity mode appears in the bulk band gap when the cavity is small (*e.g.*, when L = 2). As the cavity size increases, cavity modes begin to appear in the bulk band gap, and their number continues to increase. As an example, when L = 4, cavity modes already exist in the bulk band gap, and the first modes that appear are modes 1(1') and 2(2'), whose frequencies are not split; *i.e.*, the TI-WGMs. As the cavity size further increases to a certain extent, such as when L ≥ 6, mode 0 (or 0') and mode 3 (or 3') with split frequencies (*i.e.*, the TI-SWMs) begin to appear in the bulk band gap. As the particle size further increases, an increasing number of TI-WGMs and TI-SWMs appear, and the degree of the frequency splitting of TI-SWMs decreases.

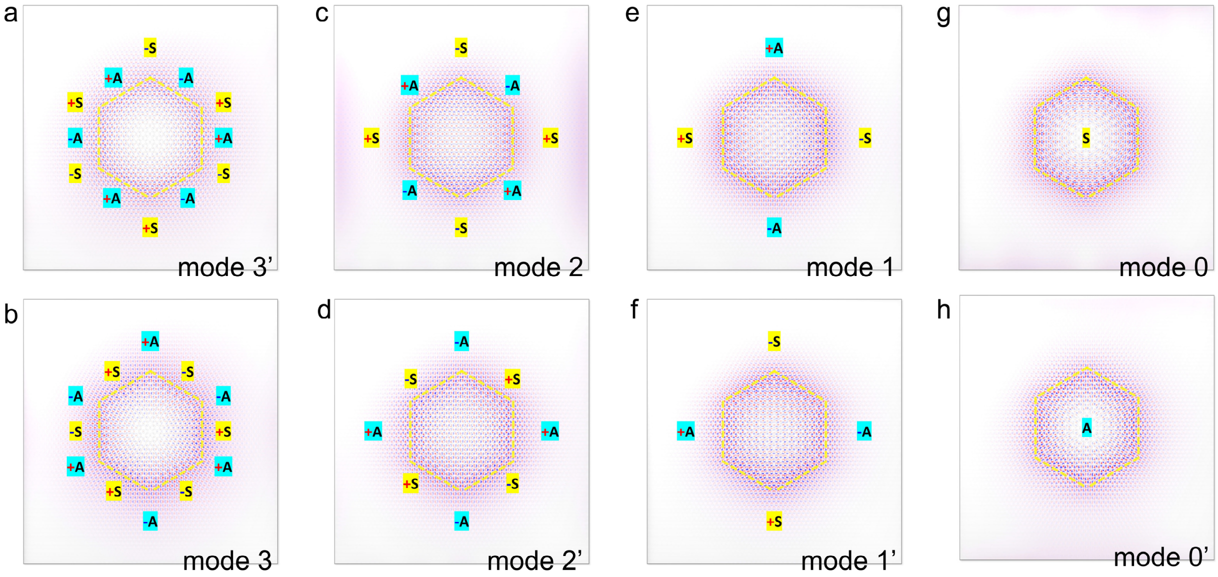

Supplementary Fig. 6 | Paired cavity modes shown in Fig. 1 of the main text.

a to **h**: Elastic field distribution of the paired modes, from low frequency to high frequency. The two modes in each pair have the same spatial rotational symmetry about the centre of the hexagon cavity. Therefore, naming these paired cavity modes according to their symmetry index, from low frequency to high frequency, as modes 3(3'), 2(2'), 1(1'), and 0(0') is advantageous.

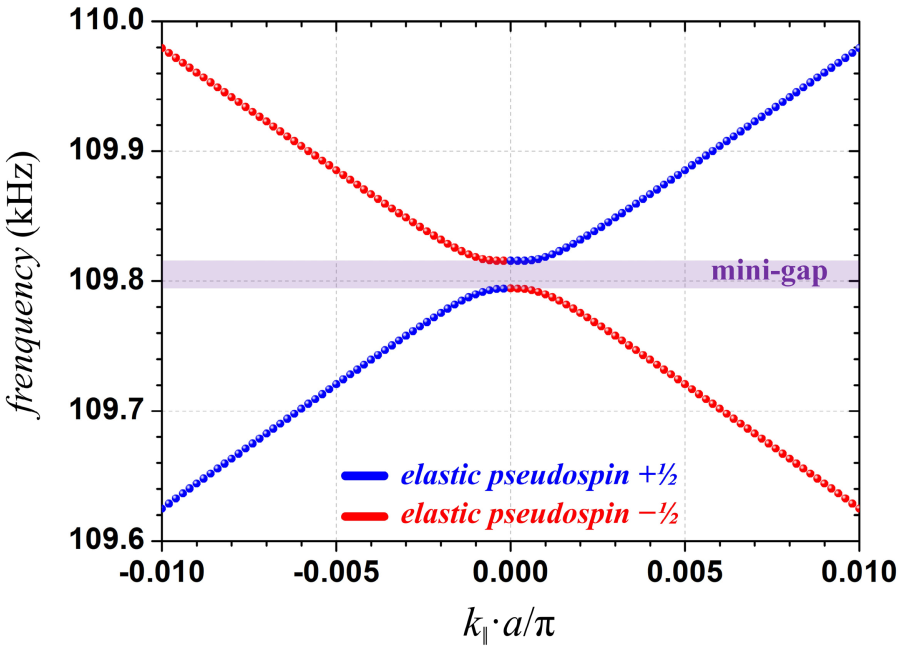

Supplementary Fig. 7 | Mini-gap of the two elastic helical edge states at $k_\parallel = 0$.

Owing to the breaking of lattice symmetry at the TI – OI boundaries, coupling between spin-up (pseudospin +½) and spin-down (pseudospin −½) is unavoidable in our system, which means that the bands of the helical edge states are supposed to have a mini-gap, as shown in the calculated projection band structure of the elastic TI–OI interface in **Supplementary Fig. 7**. However, in this system, the mini-gap is small, less than 1/500 of the entire TI band gap. It is therefore reasonable that the coupling and conversion of spin-up and spin-down in the TI waveguide may be weak.

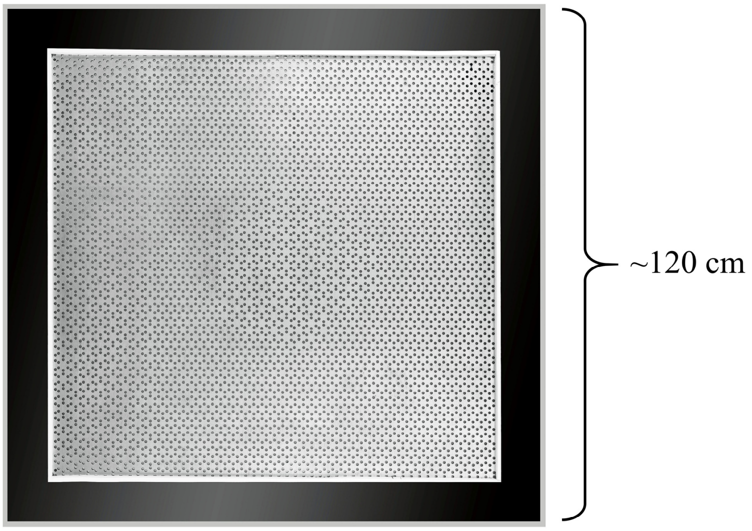

Supplementary Fig. 8 | Photograph of the front of one of our experimental samples.

Samples in this work are prepared exclusively on polished stainless-steel plates. The plates are perforated on a precision CNC milling machine to create phononic crystals with an identical hole radius r = 3.52 mm. An impedance-matched soundproof adhesive, comprising epoxy resin, graphite, and tungsten powder, is widely coated on the circumference of the samples (the black regions) to prevent unwanted solid-state acoustic reflections.

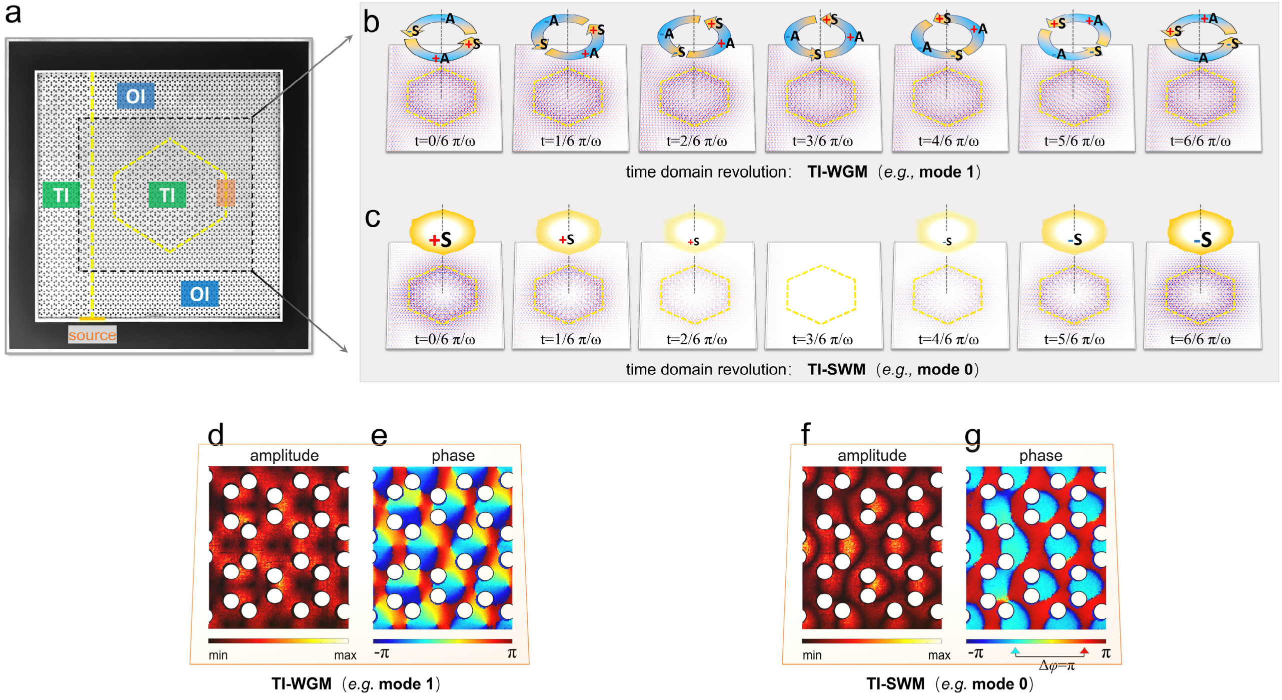

Supplementary Fig. 9 | Cavity mode excitations of two different categories.

a: Sample image and experimental configuration, similar to the cases shown in **Figs. 3** and **4** of the main text. **b**: Calculated elastic displacement field distribution of the TI cavity in the half resonance period when a TI-WGM (*e.g.*, mode 1 (1')), is excited. Elastic energy is mainly concentrated on the boundary of the TI cavity and exists in the form of a travelling wave. The elastic wave on the cavity boundary is similar to that in the case of a helical edge state with spin +½ in the straight waveguide, evolving in the form of "$+S$ — $+A$ — $-S$ — $-A$" in the time domain. S and A are the base vectors of the TI-OI helical edge states (*i.e.*, $S \pm \mathrm{i}A$ with spin ±½). **c**: Calculated elastic displacement field distribution of the TI cavity in the half resonance period when a TI-SWM (*e.g.*, mode 0) is excited. Although the elastic energy is still mainly concentrated on the boundary of the TI cavity, the elastic waves collectively oscillate throughout the cavity, including at the boundaries. **d** and **e**: Experimentally measured field distributions of the amplitude and phase, respectively, of the elastic waves inside the TI cavity (orange box shown in **a**) when mode 1 is excited. The phase changes continuously, following the characteristics of travelling waves. **f** and **g**: Experimentally measured field distributions of the amplitude and phase, respectively, when mode 0 is excited. Here, the phase change is not continuous but has an island-like distribution with a phase difference of 180°, which is a standard feature of standing waves.

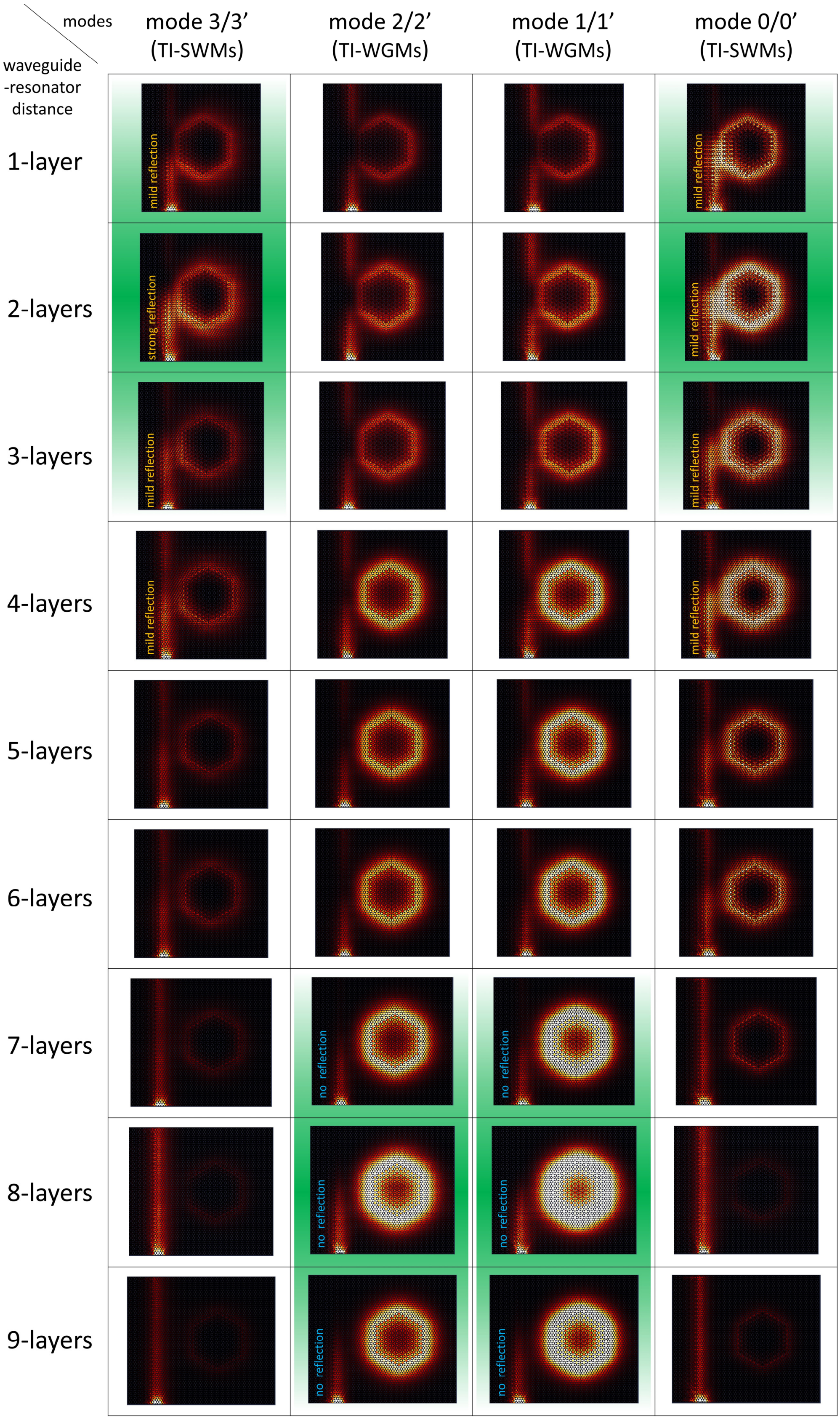

Supplementary Fig. 10 | TI waveguide-resonator distance for critical couplings. All the figures are simulated elastic energy field distributions in the resonance of four main TI cavity modes, when there is an upward incidence in the left waveguide of the TI waveguide-resonator

system. From top to bottom, the "waveguide resonator distance" is modeled as one to nine layers. Critical couplings (CCs) occur in cases inside the green boxes. In the CC situations of TI-SWMs, obvious backscattering (*i.e.*, reflection) can be observed returning to the incident port of the TI waveguide. In the CCs of TI-WGMs, by contrast, no reflection is observed.

There is a successful coupled mode theory putting forward a criterion for the critical coupling between a waveguide and ring resonator. As a classic paper [*Yariv, A. Critical coupling and its control in optical waveguide-ring resonator systems. IEEE Photonic Tech L.* ***14****, 483–485 (2002)*] points out: put simply, when a ring resonator and straight waveguide are close to each other and coupled, if the coupled resonator induced loss to the waveguide (generally referred to as **coupling loss** for short; depending on the coupling strength between the waveguide and resonator) is equal to the intrinsic loss of the resonator itself (generally referred to as **intrinsic resonator loss** for short; depending on the quality of the resonator), the transmittance of the waveguide drops to zero, and we say that the ring resonator and waveguide are critically coupled.

For the above two losses, 1) a larger distance between the waveguide and resonator results in smaller coupling loss owing to the weak coupling between them and 2) a higher quality of the resonator results in smaller intrinsic resonator loss. However, the specific values of these two losses are not directly given by theory, and the practical "waveguide resonator distance" required for critical coupling generally needs to be determined by means of experiments or at least numerical simulations.

Underlying the experiments presented in our present paper, we conducted numerical simulation to systematically investigate the distance required for the critical coupling between the TI cavity and TI waveguide. As stated in our main text, the result is that when the waveguide resonator distance is relatively **small**, TI-SWMs are prone to critical coupling with the TI waveguide; in contrast, when the waveguide resonator distance is relatively **large**, TI-WGMs are prone to critical coupling with the TI waveguide. In the main text, we simply show two representative examples, which demonstrate independently the critical coupling phenomenon of the two different types of modes; *i.e.*, the two-layer distance for TI-SWMs (mode 3/3' and mode 0/0') and eight-layer distance for TI-WGMs (mode 2/2' and mode 1/1'). In fact, according to our simulations, when the waveguide resonator distance is from one to three layers, all TI-SWMs have critical coupling with the waveguide. Additionally, for TI-WGMs, the distance ranges approximately between seven and nine layers.

Supplementary Fig. 10 shows our numerical simulations on this issue. In the simulations, the designed TI cavity is completely consistent with that in our experiment, and the waveguide resonator distance is modeled from one to nine layers. Both material intrinsic loss and in-plane radiation loss (*i.e.*, the two governing losses of our system) were considered in the simulations. These simulations investigated whether the four main modes (*i.e.*, modes 3, 2, 1, and 0) in our paper can critically couple with the TI waveguide, and whether backscattering

occurs during the critical coupling. The results are as follows.

1) When the waveguide resonator distance is from one to three layers: Only TI-SWMs (mode 3/3' and mode 0/0') are critically coupled with the TI waveguide, demonstrating **near-zero transmittance** of the waveguide, together with **obvious backscattering** observed returning along the original path of the waveguide.
2) When the waveguide resonator distance is from four to six layers: No mode is critically coupled with the TI waveguide. At the strongest resonances, the transmittance of the waveguide is always non-zero.
3) When the waveguide resonator distance is from seven to nine layers: Only TI-WGMs (mode 2/2' and mode 1/1') are critically coupled with the TI waveguide, demonstrating **near-zero transmittance** of the waveguide. This time, however, **no backscattering** is observed returning along the original path of the waveguide.

Our numerical simulations and experiments give consistent results; *i.e.*, TI-SWM resonators and TI-WGM resonators require different waveguide resonator distances for critical coupling to the TI waveguide. The former requires a smaller distance than the latter. The difference can be understood as follows.

1) The criterion for the critical coupling is that **the coupling loss** equals **the intrinsic resonator loss**.
2) **The coupling loss** is related to the waveguide resonator distance; a larger distance results in weaker coupling and a smaller loss.
3) **The intrinsic resonator loss** is related to different TI cavity modes: TI-WGM resonators are backscattering suppressed, resulting in greatly reduced loss; TI-SWM resonators, instead, bring backscattering to the coupled waveguide-resonator, and their energy is more easily consumed, leading to a much greater equivalent loss than for the TI-WGM resonators.

It is therefore reasonable that TI-WGM resonators require a larger waveguide resonator distance than TI-SWM resonators to achieve critical coupling with the TI waveguide.

Although the specific waveguide resonator distance for critical coupling can hardly be given analytically (with one needing to consider the specific physical system, mechanical dynamics, material loss, air damping, complicated structure, and other factors), numerical simulation can provide great help, even if there may be a little deviation in the result. Additionally, for macro-scale crystalline materials (*e.g.*, photonic crystals and phononic crystals), the waveguide resonator distance between our proposed TI cavity and TI waveguide always needs to be **an integer multiple of the atomic layer**. Therefore, whether for phononic or photonic TIs, it is simple and efficient to conduct numerical simulation to roughly determine the spacing (*i.e.*, number of atomic layers) where critical coupling may occur.

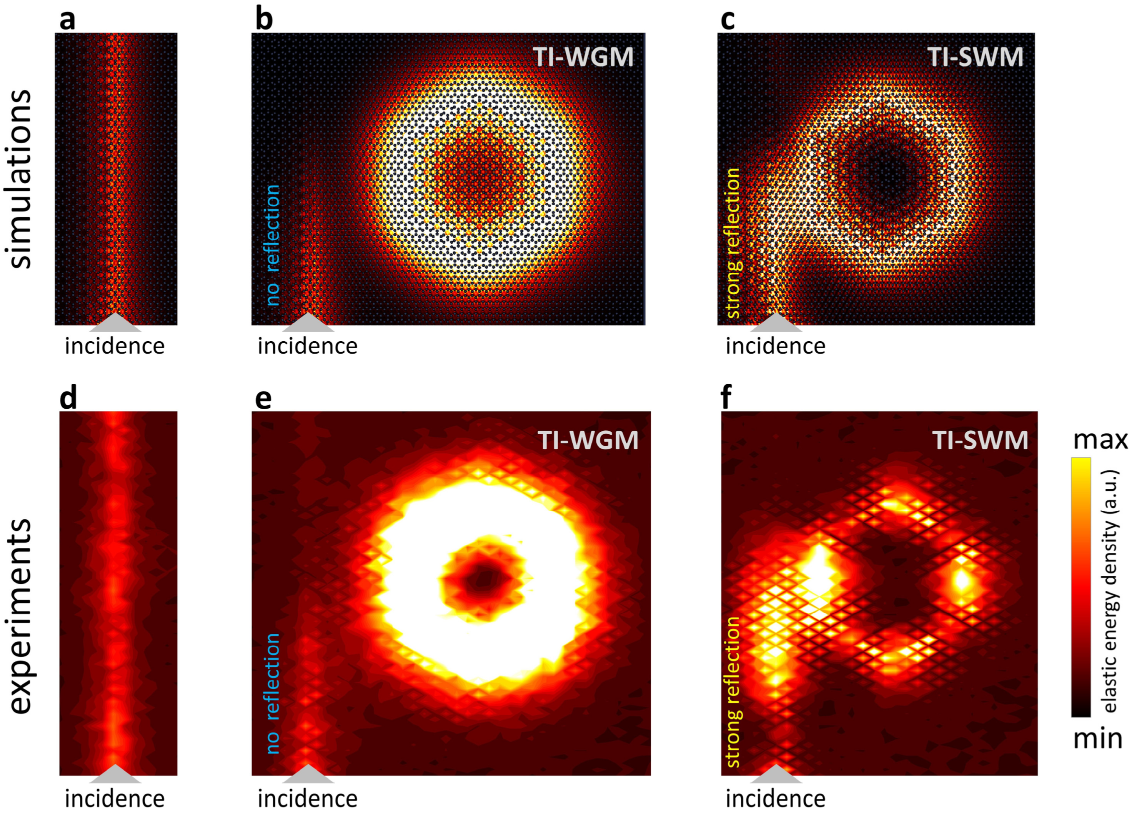

Supplementary Fig. 11 | Experimental vs. simulation results for TI waveguide-resonator critical couplings

a, **b**, and **c** are respectively simulated elastic energy densities for the TI waveguiding and critical coupling with zero transmittance in the TI-WGM case and TI-SWM case. **d**, **e**, and **f** are the corresponding experimental results, the same as in Figs. 3 and 4 in our main text. In the simulations, the out-of-plane radiation loss (*i.e.*, air damping) is set to zero. It is seen that numerical simulations are in good agreement with the experimental results. Only in the critical couplings between the TI-SWM cavity (*i.e.*, cavity working as the TI-SWM) and the TI waveguide do we observe appreciable reflectance (backscattering) back to the incident port. In all other situations, no reflectance (backscattering) is observed back to the incident port. Both results confirm that the backscattering is **essentially and only** due to the TI-SWM cavity, while the TI waveguide and TI-WGM cavity hardly bring any backscattering.
